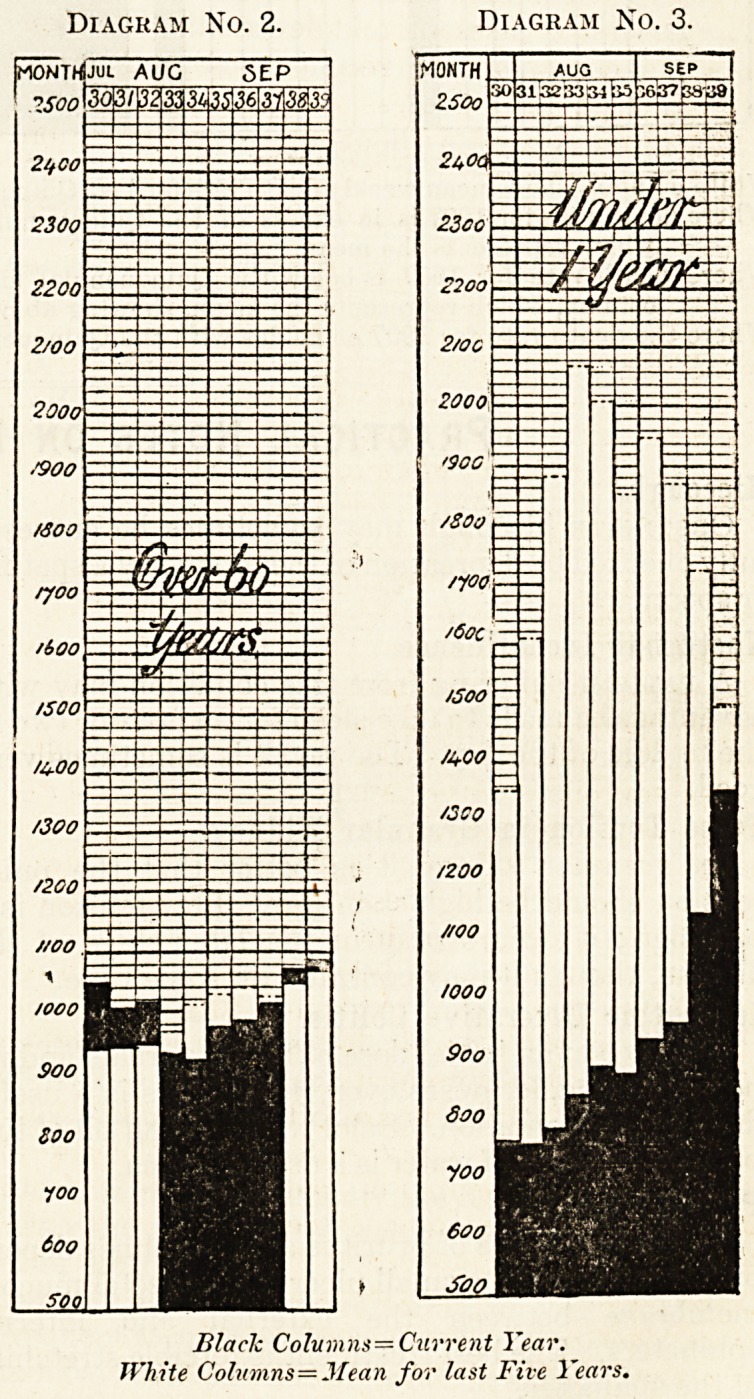# The Cold Summer and the Death Rate

**Published:** 1907-11-30

**Authors:** 


### Public Health and Hygiene.

### THE COLD SUMMER AND THE DEATH RATE.

A glance at the diagram printed on p. 242 shows the extraordinary departure witnessed this summer from the mean weekly death-rate for the last five years of the 76 great towns of England and Wales.

A departure so phenomenal, on the face of it, must necessarily have been associated with some unusual and potent condition affecting generally the urban population in this country. Such a condition is to be found in the weather experienced during what we must misdescribe as the summer of this year. There can be little question that it is to this factor we must ascribe the materially reduced mortality of the season. This will be the more obvious if we consider not alone the general death-rate charted in the diagram, but also the death-rates at extremes of age periods.

If, for instance, we take the deaths abstracted by the Registrar-General of persons aged 60 years and upwards, we find that the departure from the mean weekly deaths at this age period for the 76 great towns is practically negligible. The actual figures are shown in the following table:—

Deaths of Persons Aged 60 Years and Upwards.

| Week | 30 | 31 | 32 | 33 | 34 | 35 | 36 | 37 | 38 | 39 |
|---|---|---|---|---|---|---|---|---|---|---|
| Mean number of deaths 60 and over last 5 years | 937 | 940 | 944 | 965 | 1,008 | 1,026 | 1,040 | 1,022 | 1,045 | 1,068 |
| Do., 1907 ... ... | 1,046 | 1,004 | 1,016 | 932 | 919 | 977 | 986 | 1,018 | 1,068 | 1,086 |

Now if, on the other hand, we take the corresponding deaths of children under one year of age, a most striking departure is to be observed.

Here are the actual figures:—

Deaths of Children Under 1 Year of Age.

| Week | 30 | 31 | 32 | 33 | 34 | 35 | 36 | 37 | 38 | 39 |
|---|---|---|---|---|---|---|---|---|---|---|
| Mean number of deaths under 1 year last 5 years | 1,356 | 1,610 | 1,884 | 2,070 | 2,010 | 1,849 | 1,953 | 1,866 | 1,724 | 1,496 |
| Do., 1907 ... ... | 761 | 755 | 785 | 840 | 887 | 872 | 932 | 961 | 1,142 | 1,354 |

These results are more clearly seen when these figures are charted as in the diagrams Nos. 2 and 3. It can then be appreciated at a glance that the cold season has been responsible for an enormous reduction in infantile mortality. This reduction is a very striking fact. It represents a saving of between eight and nine thousand lives in the 76 great towns alone, chiefly from zymotic diarrhœa.

### Its Influence on Bottle-fed Babies.

The prevailing type of weather has accomplished what for several years it has been the aim of medical officers of health and others engaged in public health work to bring about. Inquiry would probably show that this saving has for the most part been effected among children artificially fed during the period when properly they should have been suckled by their mothers. Such children may be considered to be physically handicapped by this misfortune of their infancy. They will carry with them in a weakened physique this stigma of a babyhood despoiled of its natural fostering.

### Some Results.

Withal this abnormal survival of infants will materially disturb the age constitution of our population for some years to come. Owing to the accumulation of children at an age period when mortality is high the immediate effect will be a tendency to raise the general death-rate. Later on, owing to accumulation of young persons at an age period of very low mortality, the tendency will be to lower the general

Diagram No. 2.

Diagram No. 3.

*Black Columns = Current Year.*
*White Columns = Mean for last Five Years.*

death-rate; but owing to the operation of other factors and the comparative insignificance of this it will not, of course, be possible to trace these effects. But it is interesting to note that the echo of the cold summer of 1907 will reverberate through the vital statistics of the coming years.

## Figures and Tables

**Diagram No. 2. Diagram No. 3. f1:**